# Multiscale Coupling of Uterine Electromyography and Fetal Heart Rate as a Novel Indicator of Fetal Neural Development

**DOI:** 10.3389/fneur.2019.00760

**Published:** 2019-07-17

**Authors:** Kun Chen, Yangyu Zhao, Shufang Li, Lian Chen, Nan Wang, Kai Zhang, Yan Wang, Jue Zhang

**Affiliations:** ^1^Academy for Advanced Interdisciplinary Studies, Peking University, Beijing, China; ^2^Obstetrics and Gynecology, Peking University Third Hospital, Beijing, China; ^3^College of Engineering, Peking University, Beijing, China

**Keywords:** fetal neural development, uterine electromyography, fetal heart rate, cross-wavelet analysis, intrauterine growth restriction, small for gestational age

## Abstract

Fetal nerve maturation is a dynamic process, which is reflected in fetal movement and fetal heart rate (FHR) patterns. Classical FHR variability (fHRV) indices cannot fully reflect their complex interrelationship. This study aims to provide an alternative insight for fetal neural development by using the coupling analysis of uterine electromyography (UEMG) and FHR acceleration. We investigated 39 normal pregnancies with appropriate for gestational age (AGA) and 19 high-risk pregnancies with small for gestational age (SGA) at 28–39 weeks. The UEMG and FHR were recorded simultaneously by a trans-abdominal device during the night (10 p.m.−8 a.m.). Cross-wavelet analysis was used to characterize the dynamic relationship between FHR and UEMG. Subsequently, a UEMG-FHR coupling index (UFCI) was extracted from the multiscale coupling power spectrum. We examined the gestational-age dependency of UFCI by linear/quadratic regression models, and the ability to screen for SGA using binary logistic regression. Also, the performances of classical fHRV indices, including short-term variation (STV), averaged acceleration capacity (AAC), and averaged deceleration capacity (ADC), time- and frequency- domain indices, and multiscale entropy (MSE), were compared as references on the same recordings. The results showed that UFCI provided a stronger age predicting value with R^2^ = 0.480, in contrast to the best value among other fHRV indices with R^2^ = 0.335, by univariate regression models. Also, UFCI achieved superior performance for predicting SGA with the area under the curve (AUC) of 0.88, compared with 0.79 for best performance of other fHRV indices. The present results indicate that UFCI provides new information for early detection and comprehensive interpretation of intrauterine growth restriction in prenatal diagnosis, and helps improve the screening of SGA.

## Introduction

There is increasing consensus that many adverse outcomes, such as stillbirth, neonatal complications (1), and impaired neurobehavioral and motor development during childhood (2, 3), are associated with intrauterine growth restriction (IUGR), also referred to as fetal growth restriction (FGR). IUGR is defined as the pathologic inhibition of intrauterine fetal growth of the fetus that fails to reach its growth potential (4). In clinical practice, the structural parameters estimated by ultrasound is the preferred method to screen fetal developmental problems in the uterus. The IUGR referred for ultrasound evaluation, commonly determined by population standards for estimated fetal weight (EFW) <10th and/or abdominal circumference (AC) <10th *in utero* (5). Similarly, small for gestational age (SGA) is most commonly defined as a birthweight below the 10th percentile for the gestational age in the newborn (5). They both represent a condition that, in the context of fetal development, may serve as a model of possible delay of structural parameters due to chronic nutritional deprivation and hypoxemia. Many studies have shown the possible delay in the functional maturation of the sympathetic nervous system (related to FHR accelerations) (6, 7).

Movement-related heart rate acceleration patterns monitored by electronic fetal monitoring (EFM) may provide additional information for fetal neurodevelopment.

Recently, a series of studies based on fetal magnetocardiographic (fMCG) with high temporal resolution demonstrated that fHRV patterns directly reflected the development and maturation of the fetal nervous system. Van Leeuwen and co-workers (8) noted that a complexity measure of RR intervals increased linearly with fetal age (R^2^ = 0.79). Similarly, Hoyer et al. (9) demonstrated that the multiscale entropy (MSE) of FHR over a range of short scales increased with age in the quiet state, and age dependencies were found to be weaker in the active state. Further, Hoyer et al. developed a fetal autonomic brain age score (fABAS) based on various fHRV indices and achieved excellent performance (10, 11). However, the relevant indicators are based on the RR sequence of fMCG, and their analysis from common 4 Hz resampled FHR is pending. In addition, several topical fHRV indices, short-term variation (STV) (12–14), averaged acceleration capacity (AAC) and averaged deceleration capacity (ADC) based phase-rectified signal averaging (PRSA) methodology (15–17), and MSE (18) are commonly used indicators for screening IUGR and SGA. However, such studies were limited by a single short-term FHR signal and might be easily affected by other factors including sleep cycle and women's status (19, 20).

Another core of assessing fetal development lies in the emergence of a temporal association between fetal movement (FM) and FHR acceleration (21, 22). Previous studies empirically determined a coupling, depending on the fixed amplitude and interval time. Moreover, FM-FHR coupling is quantified by cross-correlation (23), but the effectiveness of cross-correlation is limited by non-stationary signals, and mainly, it is not suitable for the analysis of long-term FHR data that might evolve and become drastically different over time. Currently, significant progress has been made in long-term FHR and fetal movement-related uterine electromyography (UEMG) monitoring technology based on abdominal electrical signals (24). Meanwhile, new coupling analysis methods based on wavelets have achieved impressive performance in evaluating the dynamic properties of cerebral autoregulation in autonomic failure patient, and neonatal hypoxic-ischemic encephalopathy during hypothermia (25). The wavelet technique has several advantages: it makes no assumption about the stationarity of input signals (26), and the cross-wavelet power spectrum can characterize time-varying common power between two signals at multiple scales of frequency (27, 28).

In this study, we use the cross-wavelet power spectrum to quantify the coupling of UEMG signal and FHR accelerations. We postulate that the coupling extent could provide new information for fetal neurodevelopment, and help improve the surveillance of SGA fetuses. Also, the performances of classical fHRV indices, including short-term variation (STV), averaged acceleration capacity (AAC), and averaged deceleration capacity (ADC), time- and frequency- domain indices, and multiscale entropy (MSE), were compared as references on the same recordings.

## Methods

### Study Design and Population

This study was approved by the Ethical Committee of Peking University Third Hospital. Each subject signed an informed consent before enrolling.

From June 2014 to 21 November 2018, we recruited high-risk pregnancies with hypertensive disorders complicating pregnancy (HDCP) and/or IUGR between 28 and 39 weeks gestational age. Also, 19 pregnancies with SGA newborns were included in the study group. The controls were 39 uncomplicated pregnancies with newborns whose birthweight were appropriate for gestational age (AGA), (See the next section for a detailed definition of HDCP, IUGR, and SGA). The following conditions served as exclusion criteria—maternal: multiple pregnancies, known uterine contractions during the recording; Fetal: known fetuses with chromosomal or structural anomalies, fetal cardiac arrhythmias. The information about each woman, the characteristics of the fetus and newborn outcomes were collected from the medical records.

### Maternal and Infant Characteristics

The detailed maternal and infant characteristics of the AGA and the SGA are presented in Table 1. These two groups were broadly comparable in terms of maternal age, BMI, gestational age at monitoring, and gender distribution. However, the birthweight was significantly lower in the SGA group (*P* < 0.001) compared to the AGA. In addition, from the “time internal between monitoring and birth” item, we can see that the SGA fetus were born about one month earlier than controls. Also, 42.1% (8/19) of the SGA group are preterm labor and require neonatal intensive care unit (NICU) admission in the SGA group.

**Table 1:** Summary statistics for pregnant women's and fetal demographic and characteristics between the AGA and the SGA.

	AGA group (*n* = 39)	SGA group (*n* = 19)	*P*-value
Age (years)	30.3 ± 3.5	34.6 ± 3.7	<0.001
BMI	27.1 (25.6,28.9)	27.3(24.1,29.2)	n.s.
Systolic blood pressure	121 (114,126)	130(119,141)	0.006
Diastolic blood pressure	73 (68,80)	82 (70,90)	0.019
Gestational age at monitoring (weeks)	36 (33,38)	36 (35,37)	n.s.
Gestational age at birth (weeks)	40 (39,40)	37 (35,38)	<0.001
Time interval between monitoring and birth (days)	19 (7,44)	7 (7.7)	<0.001
Gestational hypertension disease	0 (0)	13 (68.4)	<0.001
IUGR/FGR	0 (0)	9 (47.4)	<0.001
Mode of delivery			n.s.
Vaginal	27 (69.2)	9 (47.4)	
Cesarean	12 (30.8)	10 (52.6)	
Preterm labor	0(0)	8(42.1)	<0.001
Birthweight (g)	3378 ± 413	2070 ± 334	<0.001
Birthweight <10th percentile	0 (0)	19 (100)	<0.001
Birthweight <3th percentile	0 (0)	11 (57.9)	<0.001
Neonatal sex (male)	21 (53.8)	10 (52.6)	n.s.
APGAR <7	0 (0)	0 (0)	–
NICU admission	0 (0)	8 (42.1)	<0.001

*Data are mean ± std, median (quartile 1, quartile 3), or n (%) unless otherwise specified*.

### Clinical Definitions

In this study, the diagnostic criteria of HDCP in pregnancy refers to systolic blood pressure ≥140 mmHg and/or diastolic blood pressure ≥90 mmHg on at least two occasions at least 4 h apart after the 20th week of gestation. The diagnosis of IUGR was defined by both abdominal circumference and estimated fetal weight (EFW) ≤10th percentile for the gestational age at the time of mid-gestation ultrasound scan. The diagnosis of SGA was defined by a birthweight below the 10th percentile for the gestational age in the newborn. A newborn whose birthweight was appropriate for the gestational age, at 10–90th percentile, was defined as AGA (29).

### Data Collection

For each participant, UEMG and FHR were trans-abdominally recorded by the maternal-fetal monitor, Monica AN24 (Monica Healthcare, Nottingham, UK), during one night after recruitment. Note that the sampling frequency of abdominal fetal ECG is 300 Hz. Fetal heart period was determined to an accuracy of 3.3 ms as the time between consecutive QRS complexes. However, limited by the common settings of existing commercial electronic fetal monitoring (EFM) devices, we used resampled 4 Hz data for later offline analysis. UEMG signal mainly reflects fetal movement information ranges between 0 and 100 in arbitrary units (a.u.). In the actual acquisition, some signals were dropped due to the electrode slice becoming loose or the signal being masked by noise, which were set as 0. For each subject, the recording between 22:00 pm to 8:00 am, mostly overnight, was selected for analysis to minimize motor activity that could result in signal loss (24). We provided a brief summary of statistics for data characteristics between the AGA and the SGA in Table 2. The signal loss rate was the proportion of 0 bpm of the FHR value in the recording. We also used the term recording quality (RQ) during an hour to indicate a valid 60-min segment with RQ >60% based on Dawes-Redman criteria (30). As Graatsma et al. (24) reported, the data quality was satisfactory at night (10 p.m.−8 a.m.) with a loss rate of 10.86 ± 9.78 in AGA group and 16.11 ± 16.96 in SGA group.

**Table 2:** Summary statistics for data characteristics between the AGA and the SGA.

	AGA group (*n* = 39)	SGA group (*n* = 19)	*P*-value
Monitor time (hours)	9.67 ± 0.69	9.78 ± 0.59	n.s.
Signal loss (%)	10.86 ± 9.78	16.11 ± 16.96	<0.01
60-min segments	9 (8,10)	6 (6,10)	<0.01
Quiet segments	8 (5,16)	–	–
Active segments	34 (28,39)	–	–

### Data Preprocessing

The FHR preprocessing stage contains four steps: remove signal loss artifacts, evaluate the FHR baseline, eliminate FHR outliers, and extract the positive part above the FHR baseline.

**Figure 1:**
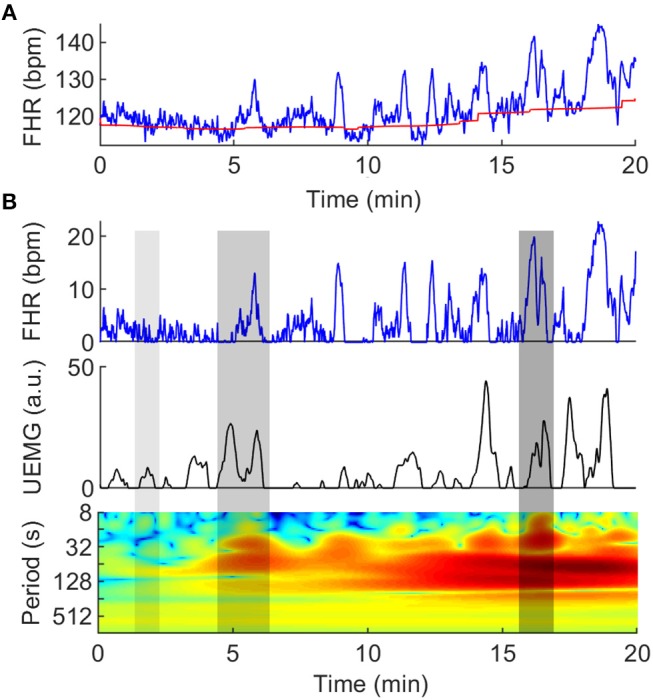
Quantification of couplings between UEMG and FHR. **(A)** Twenty-minute digital UEMG and FHR synchronous monitoring data. The top plot shows FHR (blue line), and the slow change over time is FHR baseline (red line). The second plot shows only the positive part of the FHR after removing the baseline drifts. The last plot provides UEMG data in arbitrary units. **(B)** The cross-wavelet power spectrum of the UEMG and FHR. The x-axis represents time, the y-axis represents period (1/8-1/1024 Hz), and the color scale represents the magnitude of power with a log2 scale (-10 - 10 a.u.). The three gray shadow regions (left -> right) represent almost uncoupling, narrow-scale coupling and broad-scale coupling, respectively.

1. Remove signal loss artifacts. Empirically, we have observed that most of the signal loss occurs over a continuous period of time. Thus, the part of signal loss, both in FHR and UEMG, were removed directly rather than interpolated.
2. Evaluate the FHR baseline. Baseline estimation is a necessary condition for identifying FHR acceleration and deceleration. In this study, FHR baseline is estimated by a conventional algorithm (31), which was constructed by a lowpass filter and a trim function. As the top plot in Figure 1A, the red slow change trend over time is FHR baseline.
3. Eliminate FHR outliers. There are a few outliers in FHR time series. We used the linear spline interpolation method implemented in Matlab to replaced outliers with values >25% of the baseline.
4. Extract the positive part above the FHR baseline. Considering that this study is concerned with the coupling of fetal heart rate acceleration and UEMG, we extracted the positive part above the FHR baseline for next coupling analysis (see Figure 1A).

### FHR Segmentation

From the whole night recordings, 60-min segments were selected by non-overlapping sliding window. Also, the valid segments (RQ > 60%) were included in the analysis. All 10-min segments of the recordings in AGA were selected by two independent obstetricians according to quiet and active sleep related FHR patterns. Moreover, the detailed criteria for distinguishing quiet and active states are referred to (10, 11, 32). In Table 2, we show the statistical results of the sleep states. Notably, because the status of some SGA fetuses is difficult to distinguish by the characteristics of FHR, we only consider indices based on the overall data.

In addition, when calculating the fHRV parameters for each segment (whether 60-min or 10-min), the preprocessing steps only include two steps: remove signal loss artifacts and eliminate FHR outliers. For each individual, the mean of the parameters of multiple segments is the corresponding index.

### UEMG-FHR Coupling Index

Cross-wavelet analysis, which is a time-frequency domain approach, was used to characterize the dynamic relationship between UEMG and FHR. The UEMG-FHR coupling was quantified by cross-wavelet power spectrum. A typical example of coupling analysis is shown in Figure 1B.

Briefly, the cross-wavelet power spectrum is based on the continuous wavelet transform (CWT) (26). Here, the mother wavelet is Morlet wavelet (withω0 = 6), of which Fourier period is almost equal to the scale. The CWT decomposes a signal x (n) of length N into a set of sinusoidal oscillations with specific amplitudes and phases at each frequency, which is defined as:

(1) $$\begin{array}{l} W^{\text{X}}\left(\text{n},s\right)=\sqrt{\frac{\Delta t}{s}}\overset{N}{\underset{n^{\prime }-n}{\sum }}x\left(n\right)*\left[\left(n^{\prime }-n\right)\left(\frac{\Delta t}{s}\right)\right] \end{array}$$

where n is a time index, Δt is a time step, s denotes the time scale that is in inverse proportion to frequency, and ^*^ indicates the complex conjugate. We analyzed the FHR and UEMG variability by CWT, respectively.

In order to quantify the energy of UEMG and FHR acceleration, respectively, two new parameters, the integral area of FHR power spectrum density (FHR_IAP_) and integral area of UEMG power spectrum density (UEMG_IAP_) were extracted as follows:

(2) $$\begin{array}{l} {\text{UEMG}}_{IAP}=\frac{1}{N}\overset{1/15}{\underset{1/600}{\int }}\overset{\text{N}}{\underset{1}{\int }}\;|\;W^{X}\left(n,s\right){|}^{2}dtds \end{array}$$

(3) $$\begin{array}{l} {\text{FHR}}_{IAP}=\frac{1}{N}\overset{1/15}{\underset{1/600}{\int }}\overset{\text{N}}{\underset{1}{\int }}\;|\;W^{Y}\left(n,s\right){|}^{2}dtds \end{array}$$

Where UEMG_IAP_ and FHR_IAP_ were first calculated as the mean power over time at different scales, and then the integral area of the mean power curve in a specific frequency range was calculated. Note that the specific range of s (1/600–1/15 Hz) is the frequency band of FHR accelerations (33).

The cross-wavelet power spectrum of UEMG and FHR, x (n) and y (n), is defined as:

(4) $$\begin{array}{l} W^{X\text{Y}}\left(n,s\right)=W^{X}\left(n,s\right){W^{Y}}^{*}\left(n,s\right) \end{array}$$

The |*W*^*XY*^(*n, s*)|^2^ exposes UEMG-FHR common power at a given frequency in a given time. As Figure 1B, the three gray shadow regions (left -> right) represent almost uncoupling, narrow-scale coupling and broad-scale coupling, respectively.

Also, the color scale represents the magnitude of power with a log2 scale (-10 - 10 a.u.).

We further defined an index, UEMG-FHR coupling index (UFCI), which was extracted as follows:

(5) $$\begin{array}{l} \text{UFCI =}\frac{1}{N}\overset{1/15}{\underset{1/600}{\int }}\overset{\text{N}}{\underset{1}{\int }}\;|\;W^{XY}\left(n,s\right){|}^{2}dtds \end{array}$$

Where UFCI was first calculated as the mean power of *W*^*XY*^(*n, s*) overtime at different scales, termed UFCI_S_, and then calculated the integral area of the UFCI_S_ in a specific frequency range. Essentially, UFCI is the average of UEMG-FHR multi-scale coupling energy over a period of time. In this study, a MATLAB-based software package (27) was used for wavelet analysis between UEMG and FHR.

### Representative fHRV Indices

#### CTG Compatible Indices

- STV: mean difference between consecutive R-R interval epochs in all analyzable 1 min sections. The algorithm described in Pardey et al. (30) was used to calculate STV, which first discards minutes that contain >50% signal loss or a deceleration. Then, it calculates the difference between the average pulse interval values for adjacent 3.75 second-epochs in each minute. Lastly, the values for each minute were averaged over the whole reading to give the STV.

#### PRSA Indices

- AAC/ADC: PRSA-based method calculates not only the variation of the FHR but also the speed of changes in FHR, which allows separate characterization of the average acceleration (AAC) and deceleration (ADC) capacities (15, 17). Here, the following parameters were used for PRSA: s = 10 samples, T = 10 samples, L = 50 samples; anchor points were defined as increases/decrease of <5%.

#### Time Domain Indices

- Skewness: a measure of the asymmetry of the distribution of FHR series.
- pNN5: percentage of differences between adjacent NN intervals that are >5 ms. pNN5 measures fast vagal rhythms that are reflected in the differences of successive NN intervals exceeding 5 ms.
- AC/DC: an acceleration (AC) is defined as an increase in FHR for >15 s with a minimum deviation from FHR baseline exceeding 10 bpm. A deceleration (DC) is defined as a decrease below the FHR baseline for >30 s and a deviation >20 bpm, or below the FHR baseline for 60 s and a deviation >10 bpm, respectively (30). Notably, an index with *w/o DC* (e.g., skewness w/o DC) indicates the index under exclusion of DC. Similarly, an index with *basic* (e.g., pNN5 basic) indicates the index under exclusion of DC and AC.

#### Power Spectra Indices

- VLF/LF: the ratio of very low frequencies fluctuations (0.02–0.08 Hz) compared to low frequency (0.08–0.2 Hz) band power. VLF/LF reflects the short-range FHR baseline fluctuation, according to David et al. (34).

#### Complexity Indices

- MSE: multiscale entropy (MSE) is introduced by Costa et al. (35), which extends sample entropy and investigates complexity in FHR series at multiple (time) scales.
  Here, MSE was calculated using the code provided by Physionet (https://archive.physionet.org/physiotools/mse), with embedding dimension: m = 2, tolerance level: r = 0.15, scale: 1–10.

### Statistical Analysis

For each statistical analysis, the normality of data was tested using the Kolmogorov-Smirnov test to determine whether parametric or non-parametric tests were required. All parameters of the normal distribution are expressed by mean ± std, and the independent *T*-test was used. Also, all parameters of the non-normal distribution are expressed by median (quartile 1, quartile 3), the non-parametric Mann–Whitney *U*-test was used. Moreover, the value of the different indices in predicting fetal age was assessed by univariate linear and quadratic term regression models. The coefficient determination R^2^ was used to estimate goodness-of-fit. Considering most of the predictors were significant, only non-significant results are marked by “n.s.”

Furthermore, we explored discrimination of SGA and AGA group by means of bivariate logistic regression models that include [UFCI, gestational week] and [other indices, gestational week], respectively. Receiver operator curves (ROC) were constructed from the binary logistic regression models and were compared by areas under the curve (AUC). All analyses were performed using SPSS 22 (IBM Corp, Armonk, NY). *P*-values < 0.05 were considered statistically significant.

## Results

### Normal Development of AGA

#### Whole Night Recordings

**Figure 2:**
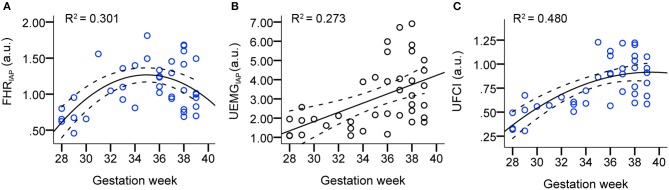
Scatter plots and linear/quadratic regression lines of FHR_IAP_ **(A)**, UEMG_IAP_ **(B)**, and UFCI **(C)** in dependency on gestation age for the AGA fetuses between 28 and 39 weeks. Note that the bold lines are quadratic mean fitted values, and dashed lines are 95% confidence intervals.

Figure 2 presents the scatter plots and linear/quadratic regression lines of FHR_IAP_ (A), UEMG_IAP_ (B), and UFCI (C) in dependency on gestation age for the AGA fetuses between 28 and 39 weeks. The parameter FHR_IAP_ clearly predicted the maturation age with a determination R^2^ = 0.301 in quadratic regression, which reflects how the FHR acceleration energy gradually increased between 28 and 36 weeks, but decreased slightly between 36 and 39 weeks. Also, the parameter UEMG_IAP_ shows an increasing trend (R^2^ = 0.273 in linear regression) with the increase of gestational age. UFCI provided a stronger age predicting value of R^2^ = 0.480 in quadratic regression. This result shows that the coupling power of UEMG and FHR acceleration is superior to their ability to predict age alone.

To comprehensively evaluate the value of UFCI for fetal neurodevelopmental age, topical fHRV indices, selected according to classical CTG (STV), PRSA-based (AAC/ADC), and fABAS-MCG related (skewness, pNN5, and MSE), were analyzed in Table 3. In univariate linear regression models, the coefficients of determination R^2^ for the model including age and fHRV indices was best for UFCI (0.449), followed by MSE10 (0.210), FHR_IAP_ (0.127) and VLF/LF (0.123). UFCI, MSE10, and FHR_IAP_ were partly improved by including a quadratic term (R^2^ = 0.480, 0.222, 0.301). However, CTG compatible STV, PRSA-based AAC/ADC, which are commonly used indicators of fetal well-being, are non-significant linear and have a quadratic relationship with gestational age. From the fABAS-MCG related time-domain parameters, pNN5 provided low univariate linear age predictors (R^2^ = 0.076), but no linear and quadratic correlation between skewness and gestational age.

**Table 3:** Analyses of whole night recordings: linear and quadratic regression models, topical indices selected according to classical CTG, PRSA-based, fABAS-MCG related, R^2^ > 0.2 in bold.

Parameter	Whole night recordings (R^2^)	
	Linear	Quadratic
**CTG compatible**		
STV (ms)	n.s.	n.s.
**PRSA based**		
AAC (ms)	n.s.	n.s.
ADC (ms)	n.s.	n.s.
fABAS-MCG related*		
**Time domain**		
Skewness	n.s.	n.s.
pNN5	0.076	n.s.
**Power spectra**		
VLF/LF	0.123	n.s.
**Complexity**		
MSE3	0.154	0.161
MSE10	**0.210**	**0.222**
**UEMG-FHR coupling**		
FHR_IAP_	0.127	**0.301**
UEMG_IAP_	**0.273**	**0.274**
UFCI	**0.449**	**0.480**

* *based on 60-min segments recordings*.

#### Quiet and Active Segments of 10 Min

Further, linear and quadratic regression analyses of fHRV indices and gestational age by 10-min segments in quiet/active sleep were shown in Table 4. In the quiet sleep segments, the time-domain indices (skewness and pNN5: R^2^ = 0.208, 0.304 in linear; 0.212, 0.304 in quadratic) were stronger univariate predictors than the complexity index (MSE10, R^2^ = 0.151 and 0.158). Concerning the exclusion of DC, the predictive value of *pNN5 w/o DC* (R^2^ = 0.333 and 0.335, linear and quadratic) and *MSE10 w/o DC* (R^2^ = 0.188 and 0.192) was increased, but skewness w/o DC (R^2^ = 0.185 and 0.186) was decreased. Concerning the exclusion of DC and AC, *skewness basic* (R^2^ = 0.255 and 0.258, linear and quadratic) was increased, but *pNN5 basic* (R^2^ = 0.194 and 0.208) and *MSE10 basic* (n.s. and n.s.) were decreased and even did not provide predictive value. In the active sleep segments, pNN5 (R^2^ = 0.161 and 0.230, linear and quadratic) predicted the fetal age, but skewness and MSE did not provide a predictive value. Overall, the predictive performances of the conventional indices (skewness, pNN5 and MSE10) were partly improved by including a quadratic term, respectively. Also, the age predicting values of these indices in the quiet stage are better than those in the active stage. However, UFCI provided a stronger age predicting value (R^2^ = 0.311 and 0.330, linear and quadratic) in the active stage in contrast to no predictive value in the quiet stage.

**Table 4:** Analyses of 10-min segments in quiet and active sleep: linear and quadratic regression models, coefficients of determination R^2^, indices selected according to fABAS-MCG and UFCI, R^2^ > 0.2 in bold.

Parameter	10-min segments (R^2^)	
	Linear	Quadratic
**QUIET SLEEP STAGE**		
**Time domain**		
Skewness	**0.208**	**0.212**
Skewness w/o DC^*^	0.185	0.186
Skewness basic^*^	**0.255**	**0.258**
pNN5	**0.304**	**0.304**
pNN5 w/o DC	**0.333**	**0.335**
pNN5 basic	0.194	**0.208**
**Complexity**		
MSE3^a^	n.s.	n.s.
MSE10	0.151	0.158
MSE10 w/o DC	0.188	0.192
MSE10 basic	n.s.	n.s.
UFCI	n.s.	n.s.
**ACTIVE SLEEP STAGE**		
Skewness^b^	n.s.	n.s.
pNN5^b^	0.161	**0.230**
MSE10^b^	n.s.	n.s.
UFCI	**0.311**	**0.330**

* *w/o DC, indices under exclusion of DC; basic, indices under exclusion of DC and AC*.

a *MSE3 has no predictive value under all conditions (quiet/active segments, w/o DC or basic)*.

b *Active sleep state*.

### Changes Associated With SGA

**Figure 3:**
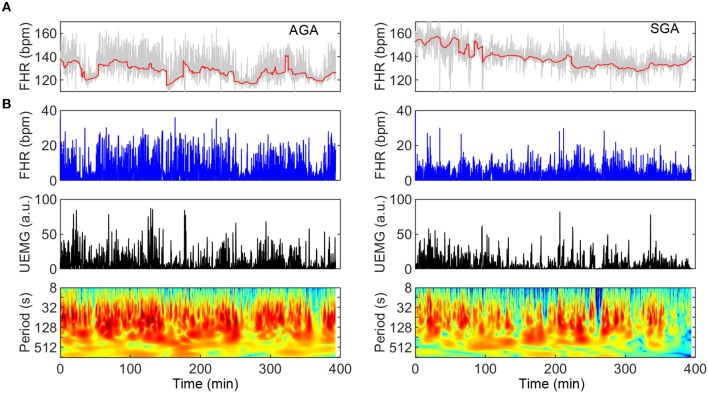
Comparison of UEMG-FHR coupling spectrums of a typical AGA fetus and an SGA fetus (left panels: AGA; right panels: SGA). **(A)** About 400-min digitized UEMG and FHR time series of two fetuses aged 35 weeks. From the FHR baseline (red line) fluctuation, the AGA fetus has an apparent sleep cycle, but SGA hardly observes it. **(B)** UEMG-FHR coupling power spectrums and corresponding time series. Note that the SGA fetus (UFCI = 0.39) result visually apparent decrease of fluctuation amplitudes of FHR and UEMG compared to the AGA result. Also, the AGA fetus (UFCI = 0.97) shows a normal pattern dominated by dense coupling, but the SGA fetus shows a high-risk pattern with sparser coupling.

SGA represents a condition that, in the context of fetal neural development, may serve as a model of delay due to chronic lack of nutritional supply. In Figure 3, two typical UEMG-FHR coupling spectrogram of an AGA fetus (UFCI = 0.97) and an SGA fetus (UFCI = 0.39) are presented, respectively. These results of the power spectrum indicate that the AGA fetus visually shows a healthy pattern dominated by dense coupling, but the SGA fetus shows a high-risk pattern with sparser coupling. In addition, from the FHR baseline (red line) fluctuations, the AGA fetus shows apparent quiet-active cycles, whereas such cycles can hardly be observed in SGA fetuses. Moreover, because the criteria previously used for dividing quiet and active stages according to FHR patterns are developed based on normal AGA fetuses, and the status of some SGA fetuses in this study are indeed difficult to distinguish, we only considered the indices based on the overall data.

**Figure 4:**
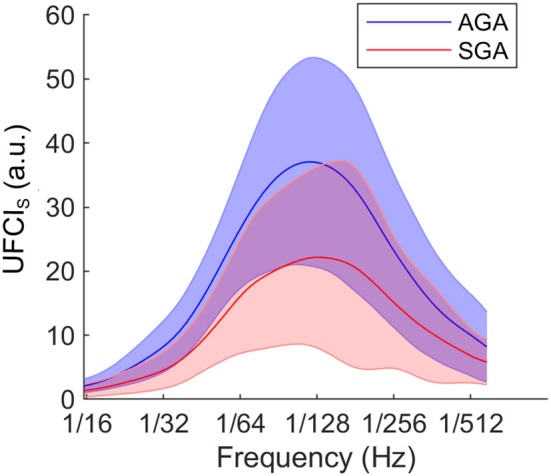
At group level, comparison of UEMG-FHR coupling power at different scales (UFCI_S_, 1/15–1/600 Hz) between the AGA and the SGA. The solid lines represent the group-averaged values, and the shaded regions denote the standard errors.

In Figure 4, we presented that comparison of UEMG-FHR coupling power at different scales (UFCI_S_: 1/15–1/600 Hz) between the AGA and the SGA at the group level. The overall level in SGA was found to be lower than that of AGA.

**Figure 5:**
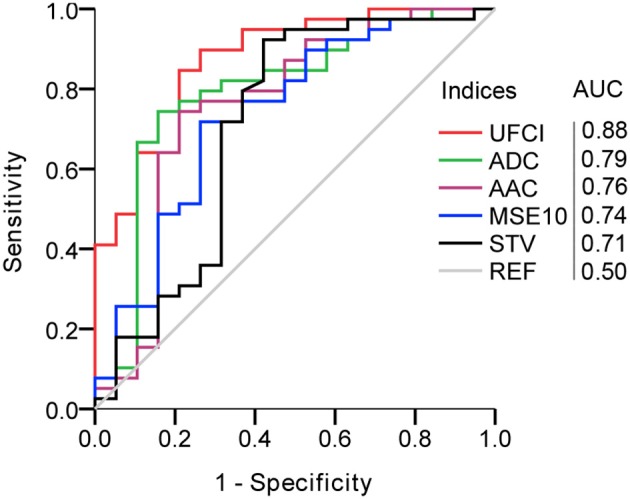
Comparison of UEMG-FHR coupling index (UFCI) and representative fHRV indices in predicting SGA. AUC, area under the curve.

In Table 5, we compared the results of representative fHRV indices, including STV, AAC/ADC, MSE10, and novel UFCI between the SGA and the AGA. The result indicates that the value of UFCI (0.47 ± 0.25) in the SGA is significantly lower (*P* < 0.01) than that of the AGA (0.78 ± 0.27). Also, the value of MSE10 (1.06 ± 0.17) in the SGA is decreased significantly (*P* < 0.01) than that of the AGA (1.20 ± 0.14). These results are consistent with the fact that SGA may serve as a model of fetal developmental delay. Additionally, the FHR_IAP_ was found to be significantly lower in SGA (0.78 ± 0.35) than that in the AGA (1.09 ± 0.35). However, there is no significant difference in UEMG_IAP_ between the SGA and the AGA. The other three fHRV indices (STV, AAC, and ADC) were all significantly (*P* < 0.01) decreased in the SGA group (8.23 ± 1.95, 7.34 ± 5.81, 3.06 ± 0.70), compared with those of the AGA control (9.69 ± 1.50, 14.2 ± 3.87, 3.84 ± 0.65). Moreover, multiple binary logistic regression was used to compare the ability of different indices to distinguish SGA. As Figure 5 demonstrates, the best single indicator is UFCI with an AUC of 0.88 (95% CI: 0.79–0.97, *P* < 0.001), compared with 0.79 for ADC (95% CI: 0.66–0.93, *P* < 0.001), and followed by AAC 0.76 (95% CI: 0.61–0.91, *P* < 0.01), and STV 0.71 (95% CI: 0.55–0.87, *P* < 0.05). Additionally, the AUC of FHR_IAP_ is 0.74 (95% CI: 0.61–0.87, *P* < 0.01) and UEMG_IAP_ provides no value for predicting SGA (95% CI: 0.42–0.75, *P* = 0.29).

**Table 5:** Summary statistics for the results of representative indices between the SGA and the AGA.

	AGA group (*n* = 39)	SGA group (*n* = 19)	*P*-value
STV	9.69 ± 1.50	8.23 ± 1.95	<0.01
AAC	14.2 ± 3.87	7.34 ± 5.81	<0.01
ADC	3.84 ± 0.65	3.06 ± 0.70	<0.001
MSE10	1.20 ± 0.14	1.06 ± 0.17	<0.01
FHR_IAP_	1.09 ± 0.35	0.78 ± 0.35	<0.01
UEMG_IAP_	3.03 ± 1.68	2.54 ± 1.60	n.s.
UFCI	0.78 ± 0.27	0.47 ± 0.25	<0.001

*Data are mean ± std*.

## Discussion

### Main Findings

In this study, a new wavelet-based approach was applied for the first time to characterize the multiscale coupling between the UEMG and FHR during long-term (>6 h) monitoring. The UEMG-FHR coupling index, UFCI was extracted from the multiscale coupling power spectrum. In univariate regression models, UFCI demonstrated a strong relationship with gestational age (R^2^ = 0.449 and 0.480, linear and quadratic, 28–39 weeks). In addition, UFCI achieved superior performance for predicting SGA with AUC of 0.88, compared with 0.79 for the best performance of other classical fHRV indices.

### Strengths and Limitations

The principal strength of the current study is the wavelet-based coupling analysis approach, which is not limited by non-stationary signals and is more suitable for processing long-term monitoring data. More importantly, our results showed that UEMG-FHR coupling index, UFCI, is superior to the single-signal energy indices (UEMG_IAP_ and FHR_IAP_), both in predicting fetal age and the SGA. Additionally, our study is based on long-term (>6 h) monitoring data, including various fetal states that can reflect more objective and sufficient fetal information. Further, the entire night's monitoring data can be transformed into the coupling spectrogram with our approach (as Figure 3), which makes it much easier to distinguish the time-varying coupling information of future long-term monitoring visually.

This study has several limitations. Firstly, the proposed coupling index, UFCI, can provide a new viewpoint on the fetal nervous development, but the small sample size and partial gestational age (28–39 weeks) might limit the identification of more subtle differences. Massive data, including more gestational age could be explored in the next. Secondly, the definition of SGA based structural parameters is a surrogate endpoint. This study did not explore the relationship between UFCI and premature delivery, or other definitions of IUGR including ultrasound blood flow parameters (16), or other adverse pregnancy outcomes. We consider that appropriately designed studies should be performed to confirm these hypotheses. Thirdly, since the presented methodology is based on FHR acceleration and fetal movement recorded by UEMG signal during antenatal, it is not applicable in the presence of fetal cardiac arrhythmias, and occurring uterine contractions during labor stages. In addition, for a fair evaluation of the here proposed coupling analysis methodology and other fHRV indices, the search for the respective optimal parameters should also be taken into consideration. This study aims to provide additional information for fetal neurodevelopment from the perspective of the coupling analysis of FHR and fetal movement.

### Interpretation

#### Predicting Fetal Neural Development

Human neuromaturation is a dynamic process, which is closely related to the fetal functional development of the central nervous system (CNS) (36). Just as fetal respiration is necessary for normal lung development, the fetal movement promotes the normal development of the limbs and the formation of specific FHR patterns closely related to CNS.

Compared with the fHRV indices (skewness, pNN5, VLF/LF, and MSE) extracted from high-precision FMCG signal, our results show a lesser performance in predicting gestational age with a decrease in R^2^, or even no predictive value (see Tables 3, 4). We think there are two main reasons for this. On the one hand, the gestational age ranged from 28 to 39 weeks in this study, but it was between 22 and 39 weeks in previous studies. On the other hand, the heart rate data used in this study was commercially available 4 Hz sampling, but previous studies used real beat-to-beat signals (9–11). For resampled FHR series, Gonçalves et al. (37) found that fHRV indices with 4 Hz sampled signals were significantly different from those indices with beat-to-beat signals. However, these differences did not affect the direction of change trend of the fHRV indices (time–and frequency–domain indices and entropy) to change with physiological changes. In our results, pNN5, VLF/LF, and short scale complexity (MSE3 and MSE10) increased with progressing gestation, which was in line with previous findings (9–11). Skewness has undergone a fundamental change with no age predicting value in 60-min segments and 10-min active segments. This is most likely due to the fact that the signal resampled by interpolation disturbs the original asymmetry. Besides, the results showed that there was no linear or quadratic correlation between STV and gestational age. This differs from the previous findings that STV describes fetal maturation over gestation (R^2^ = 0.20 and 0.21, linear and quadratic) (11). This inconsistency may be due to the heterogeneity among different data sets. A possible factor is diurnal rhythms, which could significantly affect the value of STV (38). Also, different populations and different behavioral states are also potential influencing factors.

In Figure 2C, the proposed UFCI gradually increases between 28 and 36 weeks. This characteristic curve is in line with the previous observations, which showed that the course of pregnancy between 30 and 35 weeks is an important period of autonomic nervous system maturation (39). Also, UFCI may reflect the saturating maturation after 36 weeks with a stable line between 36 and 39 weeks. Interestingly, UFCI provided a stronger age predicting value (R^2^ = 0.449 and 0.480, linear and quadratic) in the entire recording in contrast to predictive value (R^2^ = 0.311 and 0.330, linear and quadratic) in active segments. This result may be explained by the fact that UFCI of the entire recording contains more information than that of the active stage. Specifically, UFCI of the entire recording contains information on two aspects: one is the coupling energy of each time point in the active state, and the other is the ratio of duration time of the active state to total monitoring time (RAS) that has been shown to provide predicted value for fetal development (R^2^ = 0.190 in quadratic regression). In other words, UFCI reflects the information of sleep cycles (cycling of quiescent and active states, see Figure 3, left panels: AGA), which is valuable information reflecting the maturity of the nerves (40).

In addition, because the active stage includes most of movement-related FHR accelerations, it is easy to infer that the value of UFCI in the active state is significantly higher than that in the quiet state (see Figure 3, left panels: AGA). In future investigations, we will evaluate the performance of UFCI in distinguishing quiet and active states under the reference of gold standard (such as ultrasonic testing).

#### Predicting the SGA

For predicting the SGA fetus, our findings based on representative fHRV indices broadly support previous works in this area (see Table 5), including decreased STV (12–14, 40), decreased AAC and ADC (16, 17), and decreased MSE10 (18). AAC and ADC are superior to STV, and this result is consistent with previous studies (16, 17). In addition, the performances of all fHRV indices (STV, AAC/ADC, and MSE10) in screening SGA were worse than UFCI. Moreover, UEMG_IAP_ and FHR_IAP_ performed worse than UFCI in predicting fetal developmental age and screening for SGA.

This indicates that coupling analysis with the combination of FHR and UEMG information could lead to better performance.

The main clinical manifestations of SGA are malnutrition and hypoxia. Previous studies have shown the possible delay in the functional maturation of the sympathetic nervous system (related to FHR accelerations) due to chronic nutritional deprivation and hypoxemia (6, 7). Besides, it is believed that if the fetus is not supplied with enough oxygen through the placenta, it will often respond by reducing exercise (6, 7). This also means that the SGA fetus may have a less active status and lacks a sleep cycle. These may be the main reasons why UFCI was significantly (*P* < 0.01) decreased in the SGA group compared with those of the AGA control. Moreover, the SGA predicted by neurodevelopmental indicators (UFCI and other fHRV indices) was slightly different from SGA defined by birth weight ≤10th, which may imply that those were constitutionally small but showed normal neurodevelopment. Also, the SGA with premature delivery may be associated with accelerated and altered nerve maturation.

## Conclusion

Our study proposed a novel indicator UFCI from the perspective of the multiscale coupling analysis between UEMG fluctuation and the associated FHR acceleration.

UFCI provided a stronger age predicting value of R^2^ = 0.480 in quadratic regression (between 28 and 39 weeks), in contrast to univariate regression models based on other fHRV indices. Further, we demonstrated that UFCI achieved superior performance in predicting SGA (AUC = 0.88). The present results indicate that UFCI provides new information for early detection and comprehensive interpretation of intrauterine growth restriction in prenatal diagnosis, and is helpful for improving the screening of SGA.

## Ethics Statement

This study was approved by the Ethical Committee of Peking University Third Hospital Medical Science Research, REC reference S2018231.

## Author Contributions

KC contributed to the study design, the FHR analysis, and statistical analysis, and to write the article. YW, YZ, and JZ contributed to the study design, the interpretation of the data, and writing the article. LC, SL, NW, and KZ contributed to the maintenance of the database and the fHRV analysis.

### Conflict of Interest Statement

The authors declare that the research was conducted in the absence of any commercial or financial relationships that could be construed as a potential conflict of interest.
